# *In Silico* Nanodosimetry: New Insights into Nontargeted Biological Responses to Radiation

**DOI:** 10.1155/2012/147252

**Published:** 2012-06-03

**Authors:** Zdenka Kuncic, Hilary L. Byrne, Aimee L. McNamara, Susanna Guatelli, Westa Domanova, Sébastien Incerti

**Affiliations:** ^1^School of Physics, University of Sydney, Sydney, NSW 2006, Australia; ^2^Centre for Medical Radiation Physics, University of Woollongong, Wollongong, NSW 2522, Australia; ^3^Department of Physics, Technical University of Munich, 85748 Garching, Germany; ^4^Université Bordeaux 1, CNRS/IN2P3, Centre d'Etudes Nucléaires de Bordeaux-Gradignan, 33175 Gradignan, France

## Abstract

The long-held view that radiation-induced biological damage must be initiated in the cell nucleus, either on or near DNA itself, is being confronted by mounting evidence to suggest otherwise. While the efficacy of cell death may be determined by radiation damage to nuclear DNA, a plethora of less deterministic biological responses has been observed when DNA is not targeted. These so-called nontargeted responses cannot be understood in the framework of DNA-centric radiobiological models; what is needed are new physically motivated models that address the damage-sensing signalling pathways triggered by the production of reactive free radicals. To this end, we have conducted a series of *in silico* experiments aimed at elucidating the underlying physical processes responsible for nontargeted biological responses to radiation. Our simulation studies implement new results on very low-energy electromagnetic interactions in liquid water (applicable down to nanoscales) and we also consider a realistic simulation of extranuclear microbeam irradiation of a cell. Our results support the idea that organelles with important functional roles, such as mitochondria and lysosomes, as well as membranes, are viable targets for ionizations and excitations, and their chemical composition and density are critical to determining the free radical yield and ensuing biological responses.

## 1. Introduction

The theory of radiation interactions with matter, established over the course of the last century by celebrated physicists such as Bohr [1], Bethe [2], Bethe and Heitler [3], and Fano [4], now underpins a vast range of cutting-edge technologies and applications, from high-energy particle detectors for the Large Hadron Collider, to atmospheric, space and astrophysics, to electron-beam lithography and materials analysis techniques (e.g., electron microscopy, X-ray spectroscopy). For medical applications, such as imaging and radiation therapy, electromagnetic interactions that take place in living biological systems are of paramount importance because collisions can excite and ionize the constituent molecules, leading to impaired biological function. When this occuring inside a cell nucleus, there is an increased likelihood of damaging DNA and compromising the cell's viability [5].

The physical and chemical mechanisms of radiation-induced nuclear DNA damage (i.e., strand breaks and other lesions resulting from interactions on or near DNA) have been generally well understood for several decades [6] and an extensive body of literature now exists on radiobiology [7–11]. Radiation target theory [9], in particular, has provided a successful framework for achieving the fundamental aim of radiation therapy, which is to maximise tumour cell kill while sparing normal cells, assuming that initial damage to nuclear DNA is central to the killing of a cell by reproductive cell death (i.e., mitosis inhibited by loss of large amounts of genetic material). But when electromagnetic interactions occur primarily outside the nucleus, the ensuing biological damage is poorly understood [12].

Compelling evidence for such nontargeted damage is now emerging from the increasing incidence of secondary malignancies among cancer survivors treated with radiotherapy, attributed to the unavoidable exposure of healthy tissue to low-dose radiation [13–15]. At low doses, radiation can miss nuclear DNA altogether because of the small relative volume it occupies in the cell. If damaged cells that would normally be eliminated instead escape apoptosis and undergo cell cycle division, carcinogenesis may develop due to the survival and proliferation of cells with accumulating damage or mutations. Molecular signalling pathways that disrupt cellular tissue homeostasis can give rise to both acute and late effects in normal tissue following radiotherapy [16]. Indeed, there is growing concern in particular over the low-dose radiation bath into which relatively large volumes of normal tissue are immersed in modern conformal radiotherapy delivery techniques. An increasing number of radiotherapy studies are now paying more attention to normal cells surrounding irradiated tumours [17]. However, quantitative, physically motivated models are lacking in the literature, primarily because the DNA-centric approach of classical radiobiology is no longer valid at low doses. For this reason, models predicting normal tissue complications have had limited success in describing clinically observed normal tissue reactions [18].

**Figure 1:**
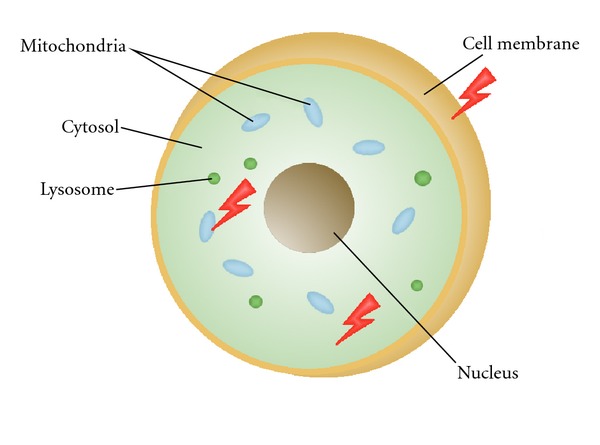
Schematic illustration of a cell, showing potentially important extranuclear targets that have been implicated in triggering complex damage signalling pathways induced by radiation (shown in red).

Further independent evidence demonstrating the limitations of the existing DNA-target paradigm for radiation-induced damage has emerged from cell irradiation experiments using microbeams, which deliver a focused beam of low-energy (typically tens of keV) radiation to spatially localised regions in a cell up to several microns away from the nucleus. These experiments provide new insights into the complex biological pathways triggered by extranuclear radiation energy deposition and resulting damage to cytoplasmic structures such as lysosomes, membranes, and mitochondria (Figure 1). Several newly recognised responses, collectively referred to as nontargeted responses, are manifested by effects such as mutagenesis (stable mutations in cell progeny), genomic instability (unstable effects caused by changes in genetic information), bystander effect (responses in neighbouring, unirradiated cells), changes in gene expression, and even adaptive responses [12, 19–24]. These effects are markedly different from the mechanistic response to DNA-targeted radiation; nontargeted responses are not directly related to the amount of energy deposited in or near the nuclear DNA of the cells traversed by the radiation. The microbeam results suggest instead that nontargeted biological responses are determined by cell's entire state, including all proteins and macromolecules in its cytoplasm, some of which may cause functional damage when released from lysosomes and mitochondria, while structural membrane damage can potentially also affect intra- and intercellular signalling pathways.

Low-energy electrons are the most abundant product of radiation interactions, and their collisions with surrounding molecules are primarily responsible for initiating the sequence of biochemical events that leads to radiation-induced damage in biological systems [25]. Much of our present understanding of radiation-induced biological damage on subcellular scales (down to nanometric volumes) stems from microdosimetry and nanodosimetry studies. A significant limitation of experimental studies, however, is that single particle detection methods rely on gas counters, which are a poor approximation to biological tissue. Nevertheless, significant progress has been made in understanding DNA damage by measuring ionization cluster-size distributions and particle tracks [26–29]. Monte Carlo (MC) simulations provide an alternate method for advancing our understanding of radiation-induced DNA damage and many independent MC models have been developed for determining DNA strand break yields by simulating electron track structure and ionization clusters in nanometric volumes or simple DNA models [30–35]. To date, however, no MC simulations have investigated the biological damage observed as nontargeted responses resulting from extranuclear irradiation.

In this paper, we briefly report *in silico* nanodosimetry studies which aim to reveal new insights into radiation-induced biological damage beyond nuclear DNA. Our simulation studies consider primary particles of different types (electrons, protons, and alphas), since nontargeted effects have been observed for radiation types of different linear energy transfer (LET) [21].  Section 2 presents the theoretical background and the MC computational approach relevant to modelling low-energy electromagnetic interactions on subcellular scales. We present and discuss our preliminary results in Section 3, and conclusions are given in Section 4.

## 2. Theoretical Background and Computational Approach

At energies below a few keV, which are relevant for electron scattering on subcellular scales, the wave properties of the incident electrons become increasingly important. Thus, the probability of inelastic scattering of incident electrons must be derived from electrodynamics rather than mechanics. Scattering cross-sections need to be derived for liquid water, which is the main constituent of biological tissue.

The Geant4 software toolkit provides state-of-the-art capability in depth and scope of Monte Carlo based approaches to modelling radiation interactions in biological systems; new models for low-energy electromagnetic cross-sections for liquid water are available for nanodosimetry and electron track structure simulations.

### 2.1. Low-Energy Electromagnetic Interactions

In the classical treatment of inelastic scattering of low-energy electrons [36, 37], the stopping power is the retarding force experienced by the incident electron due to the polarization field induced in the medium through which it propagates. The electron energy loss and momentum transfer are determined by the dielectric function *ϵ*, which describes the electromagnetic response of the medium to the disturbance caused by the incident electron. The corresponding differential cross-section for inelastic electron scattering can be expressed in terms of the inverse mean free path Λ:

(1) d2ΛdqdE=1πa0qTIm⁡[−1ϵ(q,E)],

where *T* and *E* are the electron energy before and after scattering, respectively, *ℏ* **q** is the momentum transfer, and *a*_0_ = *ℏ* ^2^/*e* ^2^ *m*_*e*_ is the Bohr radius. For electron energies large compared with atomic energies, the valence electrons can be regarded as approximately free. Using the electron plasma dielectric response for *ϵ* in (1) gives a solution consistent with the standard Bethe-Bloch stopping power derived from nonrelativistic quantum mechanics [36].

In the quantum treatment of inelastic electron scattering, the differential cross-section can be written as [2, 38, 39]

(2) $$\begin{array}{c} d\sigma =\frac{m_{e}q}{4\pi \hbar^{2}T}{\left|T_{fi}\right|}^{2}\delta \left(E+E_{n}-E_{0}\right)dq\;dE, \end{array}$$

where *E*_0_ and *E*_*n*_ are the energies of the atom before and after scattering, respectively, and *T*_*fi*_ is the transition matrix for transitions between initial and final states of the electron-atom system. The calculation of *T*_*fi*_ in the first Born approximation using an appropriate Coulomb interaction potential [38] yields

(3) $$\begin{array}{c} d\sigma =4\pi \frac{dq}{a_{0}qT}{\left|\langle n\left|d\right|0\rangle \right|}^{2}\delta \left(E+E_{n}-E_{0}\right)dE, \end{array}$$

where *d* is the matrix element of the atom's dipole moment due to the space density distribution of its electrons. The sum over all final states *n* gives the following relation for the polarizability of the atom: *Im*⁡[*ϵ* ^−1^] ≈ 4*πN*Σ_*n*_|**d**_0*n*_|^2^ *δ*(*E* + *E*_*n*_ − *E*_0_), where *N* is the number of atoms per unit volume. The relation Λ = *Nσ* then recovers (1) [36, 38, 40].

The probability of inelastic scattering of low-energy electrons, as described by (1), requires determination of *ϵ*(**q**, *E*), which has proven challenging for biological tissue. Valence electrons in soft-condensed matter cannot be regarded as free or nearly free, as they are in metals and other conductors, so the commonly used Lindhard dielectric function for an electron plasma is a poor approximation to the collective response of bound molecular electrons. Furthermore, the effective intermolecular potential in the condensed phase acts as a screen on the polarization field induced by an incident electron, and collective plasma excitations can delocalize energy deposition. The problem is further compounded by the lack of direct measurement data due to the inherent practical difficulties in conducting scattering experiments with condensed molecular targets. Although experimental data to date is only available in the optical limit (zero momentum transfer), they have nevertheless enabled improved semiempirical dielectric models to be derived and extended into the finite momentum transfer domain, *q* > 0 (i.e., finite scattering angles; e.g., [41, 42], and references therein). An alternative approach to modelling low-energy inelastic electron scattering is to use the method of partial wave expansion, which is relevant at low energies, where the Born plane wave approximation breaks down. When the incident electron energy becomes comparable to the binding energy of atomic electrons, scattering can no longer be considered a small perturbation to the system and the free electron wavefunction (i.e., a plane wave) can no longer be assumed. Champion [43] has derived new theoretical results using the partial wave approach to describe ionization of molecular water by low-energy electrons.

### 2.2. Geant4

Geant4 is an open-source software toolkit developed for general-purpose Monte Carlo radiation transport simulations [44, 45]. Its object-oriented structure and approach has enabled an impressive scope of applications, ranging from high-energy particle physics and astrophysics, to medical physics and imaging. The medical applications, in particular, are growing rapidly and are becoming more versatile; Geant4 models have been developed for radiotherapy scenarios (e.g., brachytherapy, medical linear accelerator beams, hadrontherapy, internal and radionuclide dosimetry), imaging techniques (e.g., CT, emission tomography, electronic portal imaging), and also for micro- and nanodosimetry studies, including electron track structure down to nanometric length scales. Microbeam cell irradiation experiments can also be modeled with Geant4.

In addition to the standard interaction cross-section databases for atomic collisions (i.e., NIST), Geant4 also provides additional low-energy electromagnetic classes with several choices of cross-section model data (e.g., Livermore, Penelope), including the new Geant4-DNA module (http://geant4-dna.org/), which can explicitly model all interaction events as discrete processes [46]. This extension has been specifically designed for radiobiology and nanodosimetry applications and includes implementation of a semiempirical Born model (c.f., Section 2.1) for ionization and excitation of liquid water by electrons, protons, and alphas (and a few other ions) valid for energies down to eV scales [42]. New developments currently underway include the capability to model key radiation chemistry processes such as water radiolysis.

We have been using Geant4 to develop a suite of *in silico* experiments designed to give us a better understanding of the physical processes underlying biological responses to radiation damage that occurs outside the cell nucleus. Biological damage, both structural and functional, is initiated by ionizations which produce the free radicals that have been implicated in intra- and intercellular signalling. Our models represent the first simulation studies aimed at directly addressing nontargeted biological responses triggered by radiation damage beyond nuclear DNA.

## 3. Results and Discussion

Here, we present preliminary results of three separate studies: (1) electron track structure on nanometric scales in liquid water using the Geant4-DNA semiempirical models for excitations and ionizations of low-energy electrons, (2) the spatial distribution of clustered ionization events resulting from the traversal of protons and photons through a sub-cellular scale volume of water, and (3) simulation of a microbeam cell irradiation experiment, showing localized energy deposition and ionization distributions in a realistic cell model when only the cytoplasm is irradiated by an alpha particle beam.

### 3.1. Nanometric Electron Tracks

**Figure 2:**
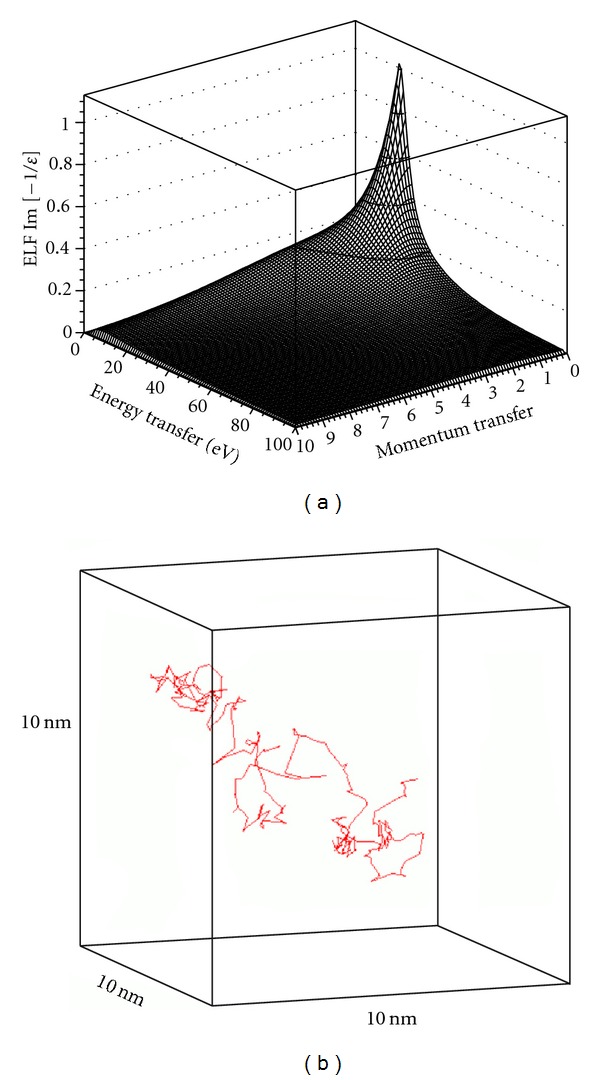
(a) Energy loss function (ELF) for low-energy electrons (c.f. (1)) in liquid water plotted as a function of energy and momentum transfer (the so-called “Bethe ridge”). (b) Corresponding track of a 100 eV electron in a 10 nm cube of liquid water.

Figure 2 shows plots of the energy loss function (ELF), *Im*⁡[−1/*ϵ*(**q**, *E*)], for low-energy electrons (c.f. (1)), and the corresponding electron track structure resulting from an electron with an initial energy of 100 eV in a 10 nm cube of liquid water. The electron ELF plotted in the energy-momentum plane, referred to as the "Bethe ridge," has been calculated for liquid water using semiempirical models for inelastic scattering [41, 42] based on those implemented in Geant4-DNA. The electron track structure has been simulated using the models for elastic and inelastic electron scattering available in Geant4-DNA (the Champion and Born models, resp.). A quantitative evaluation of these models has been presented by Incerti et al [46]. The Bethe ridge plot exhibits a prominent peak at *≃*21 eV, which is close to the plasma frequency of water, as has been noted in previous studies [41, 42]. It should be noted, however, that other inelastic channels, such as collective excitations and autoionization [47, 48], are not taken into account in this ELF. Collective excitations include plasmon-like resonances that can delocalize energy deposition away from the electron track. Autoionization is the process by which water molecules, in the liquid phase, undergo spontaneous decay. When excited by an incident electron, (H_2_O)_2_ molecules dissociate rapidly, producing secondary electrons and H_2_O^+^ ions [49, 50]. How these additional energy loss processes might change the shape of the Bethe ridge is not clear and more theoretical work is needed.

### 3.2. Ionization Cluster Distribution

In this study, we used Geant4 (version 9.4) to model monoenergetic proton and X-ray pencil beams incident on a liquid water cube, of length 40 mm, to investigate the distribution of ionization clusters and relative biological effectiveness of these different forms of radiation. The photon interactions were modelled with the low-energy electromagnetic package (based on the Livermore data libraries), including processes for the photoelectric effect, compton scattering, rayleigh scattering, and pair production. In the case of protons, the standard electromagnetic package models were used to model ionization and multiple scattering for protons with *E* ≥ 10 MeV. In both cases, protons and electrons with energies <200 MeV and <10 keV, respectively, were transported in the water medium down to a few eV, using the Geant4-DNA model extensions. The processes for electron ionization, excitation, and elastic scattering (the Champion elastic model) were included and the lower energy limit for the excitation and elastic scattering models was set to 8.23 eV [51]. The physics processes of the low-energy protons (<10 MeV) included charge decrease, excitation, and ionization [52, 53]. The simulation produced the number of ionizations in voxels of a few nanometers in size at different points along the particle trajectory (cluster size distribution).

**Figure 3:**
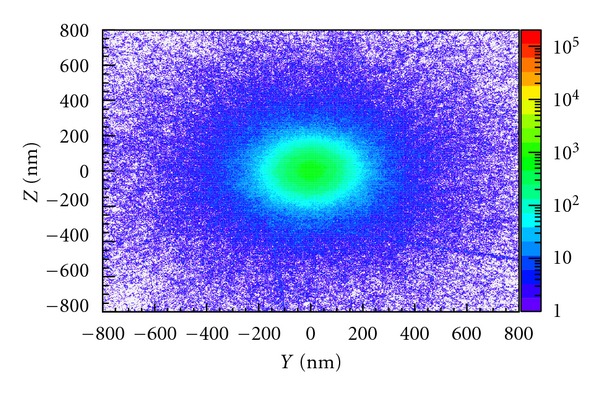
Cluster distribution of 200 MeV monoenergetic pencil beam protons in liquid water, showing the number of ionizations per 2 × 2 nm voxel at a depth of 0.25 mm.

**Figure 4:**
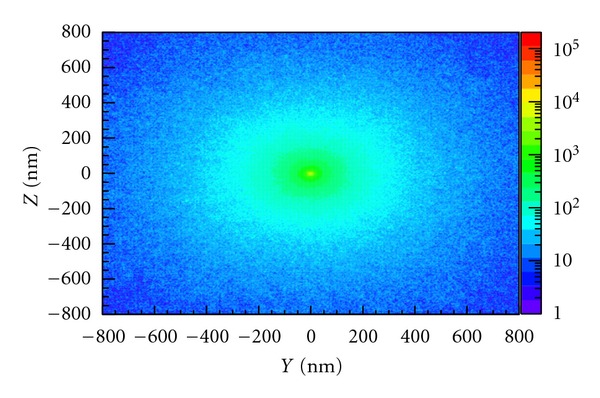
Cluster distribution of 100 keV monoenergetic pencil beam photons in liquid water, showing the number of ionizations per 2 × 2 nm voxel at a depth of 20 mm.

Figures 3 and 4 show the cluster ionization distribution of incident 200 MeV pencil beam protons and incident 100 keV pencil beam photons, respectively. The total number of each particle type was chosen to give approximately the same dose deposited in the water phantom. The number of ionizations occurring within nanometric voxels (the cluster size distribution), with dimension 2 × 2 nm, is shown at two different depths in the water phantom: 0.25 mm for protons and 20 mm for photons. In each case, the plots show the ionization clustering on the *YZ* plane. The incident pencil beam is directed along the *x*-axis and the colourbar shows the number of ionizations per voxel. For 200 MeV protons, the depth of 0.25 mm is well before the Bragg peak, but a large number of ionizations are still evident and are concentrated around the beam (c.f.  Figure 3). Photons on the other hand show very few ionizations at the same depth. At 20 mm depth, however, the number of ionizations in the vicinity of the beam is comparable to that produced by protons at 0.25 mm depth and although more scattering produces a broader spread of ionizations away from the beam, the cluster distribution around the beam axis is remarkably similar. This suggests that 100 keV photons have a similar potential to cause biological damage as 200 MeV protons.

### 3.3. Microbeam Cell Irradiation

**Figure 5:**
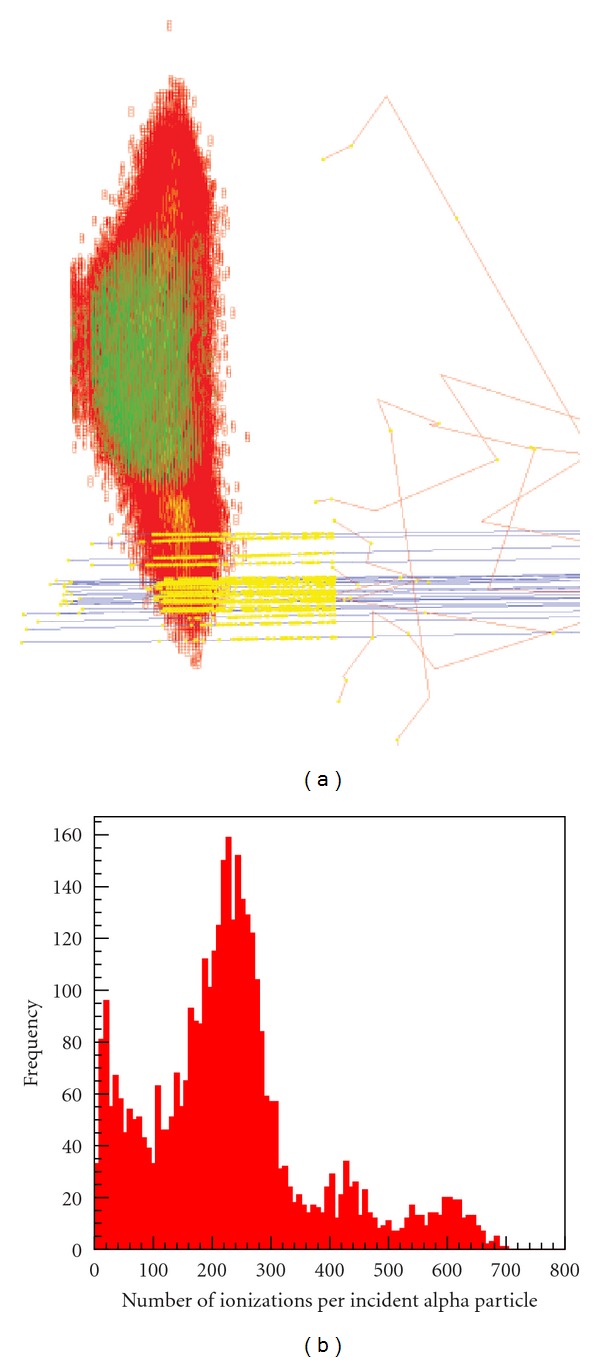
(a) Geant4 visualization of a microbeam cell irradiation simulation, showing a 5 micron beam of 3 MeV alpha particles (blue lines, incident from the right) targeting the cytoplasm (red) and avoiding the nucleus (green). (b) corresponding histogram of ionizations in the cytoplasm per incident particle for 10^4^ incident alphas.

Figure 5 shows a visualization of our microbeam cell irradiation simulation and the corresponding histogram of ionizations per incident particle in the cytoplasm. The simulation was based on the microbeam example in Geant4 (version 9.4), which is modelled on a cellular irradiation beamline facility configured to deliver a beam of 3 MeV alpha particles focussed down to 5 *μ*m in diameter. The cell geometry is a voxelized 3D model based on a human keratinocyte cell line. See Incerti et al. [54] for more details.

In our simulation, the beam was displaced off-centre, so only the cytoplasm was irradiated and not the nucleus. We used the Livermore physics processes, rather than Geant-DNA, since this allowed us to investigate the effect of chemical composition (the Geant4-DNA processes are only available for liquid water). The Livermore processes are valid down to 250 eV, corresponding to a physical scale-size ~30 nm. The cell cytoplasm and nucleus were based on a realistic chemical composition with a density of 1 g cm^−3^. The mass-fraction constituents of the cytoplasm were oxygen (*≃*58%), carbon (*≃*20%), hydrogen (*≃*9%), nitrogen (*≃*8.5%), and phosphorus (*≃*4.5%). The primary mass-fraction constituents of the nuclear material were oxygen (*≃*74.5%) and hydrogen (*≃*11%), with lower amounts of carbon (*≃*9%), nitrogen (*≃*3%), and phosphorus (*≃*2.5%) compared to the cytoplasm [55]. In addition, a number of localized overdensities (10 g cm^−3^) with the same material content as the nucleus were also distributed throughout the cytoplasm. These substructures, which could represent organelles, affect the ionization histogram (Figure 5) by reducing the average number of ionizations per event. This can be attributed to the combined effects of chemical composition and density. Despite the higher oxygen content of the substructure targets, the lower relative amounts of high atomic number elements such as carbon and nitrogen, with respect to the rest of the cytoplasm, results in an overall lower mean excitation energy and an increased probability of ionization. The higher density of the substructures increases the number of ionizations per incident alpha particle. The material density affects the overall frequency of ionizations. This result demonstrates the importance of chemical composition and density in determining the probability of ionization events and hence, production of free radical species in a real cell and subcellular structures such as organelles, membranes, as well as macromolecules and other proteins.

**Figure 6:**
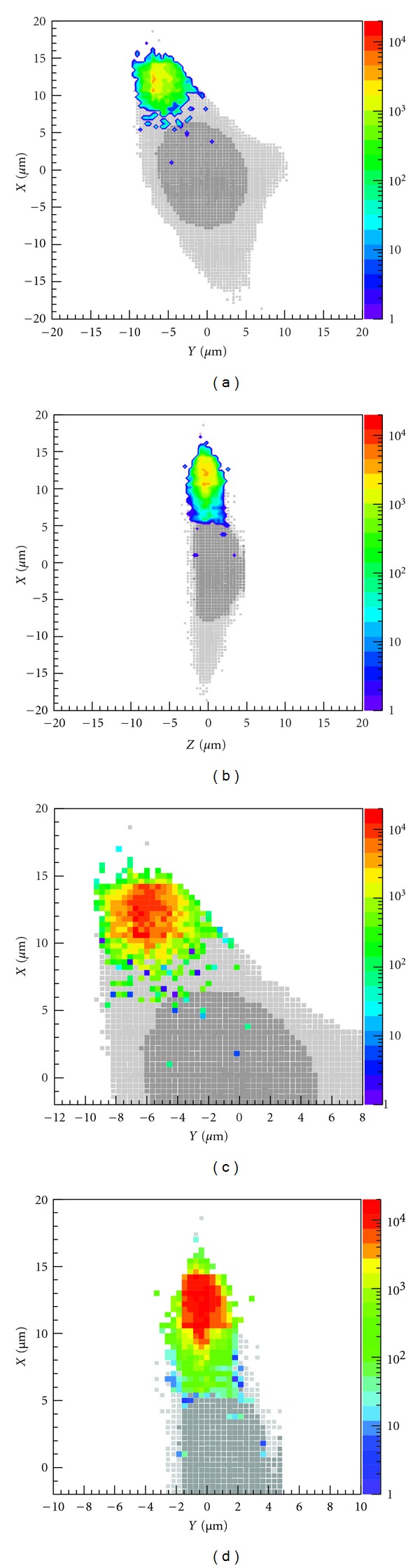
Top row: 2D projection of mean energy deposit (in eV) per voxel in the microbeam irradiated cell, showing the cross-sectional plane (left) and the longitudinal plane (right). Bottom row: corresponding distribution of number of ionizations in the cytoplasm. All plots were calculated for 10^4^ incident alpha particles.

Figure 6 shows the 2D projection of mean energy deposition per voxel and ionization events in the microbeam cell irradiation simulation. The average specific energy deposited per event is 0.08 J kg^−1^. The spatial distribution of mean energy deposition is clearly correlated with the ionization distribution in the cytoplasm. Both distributions are localized but exhibit spreading around the 5 *μ*m beam due to scattered secondary electrons. In particular, a finite number of ionizations is also found to occur along the cell and nuclear membranes, where structural damage can impair membrane-mediated inter- and intracellular signalling (i.e., via binding of ligands, diffusion of molecules through gap junctions). Within the cytoplasm, ionizations that occur in the organelles can affect the release of proteins and macromolecules, leading to functional damage. Realistic physical models for these organelles, including their spatial distribution in the cell, molecular composition, density, and size, are needed to better quantify the probability of ionizations.

A limitation of the current model is that it employs interaction probabilities based on atomic rather than molecular collisions. This means that it is not currently possible to simulate the direct production of key free radical species such as hydroxl radicals (OH^−^) and superoxide anions (O_2_^−^) which are implicated in intra- and intercellular signalling. However, a preliminary model for simulating radiolysis of molecular water and diffusion of free radical species has been implemented in the latest release of Geant4 (version 9.5). We note that the steep gradient in number density of ionizations evident in Figure 6 suggests rapid diffusion of radiolytic products across regions of the cell. We aim to explore this in future work.

## 4. Conclusions

We have presented a summary of preliminary results from a series of simulation studies investigating nontargeted responses to radiation using Geant4. Our study on low-energy electromagnetic interactions demonstrates that secondary electrons, with energies well below 100 eV, can ionize and excite liquid water on nanometric scales. More theoretical work is needed, however, to enable modelling of collective excitations and autoionization of molecular water and other molecules such as lipids which are relevant for membrane-mediated signalling. Such modelling would enable more quantitative evaluation of the likelihood of cellular dysfunction, lysis, or death resulting from primary damage to membranes and membrane-bound organelles (e.g., lysosomes, mitochondria) throughout the cell.

We also investigated the role of ionization clustering on nanoscales in liquid water without assuming *a priori* a nuclear DNA target. Our results demonstrate that low-energy photons can generate clustered ionization distributions similar to that produced by protons, suggesting a similar potential to cause biological damage. This has important implications for situations such as radiotherapy, where secondary low-energy photons are produced in abundance and can readily reach normal tissue surrounding a tumor.

Our simulation study on extranuclear microbeam irradiation of a realistic cell demonstrates the importance of chemical composition and density of cellular substructures in determining the level of radiation damage by ionizations. Although it is not yet possible to explicitly simulate within Geant4 the production of molecular free radical species that have been implicated in signalling, the spatial distribution of ionization events outside the nucleus is suggestive of the possible structural and functional damage that would trigger damage-response signalling by the cell. In future work, we plan to take advantage of the ongoing developments in Geant4, particularly the developing capability to model water radiolysis and diffusion of free radicals.
